# Majestatis Consiliarii et Archiatri, ac Medicinæ in alma et Antiquissina Vindobonensi Universitate Professoris Primarii, &c. Tomus tertius Rationis Medendi Continuatæ seu operum posthumorum Volumen I

**Published:** 1781-12

**Authors:** 


III. Antonii de Haen *quondam S. C. R. Apoſtol. Majeſtatis Conſiliarii et Archiatri, ac Medicinæ in alma et Antiquiſſina Vindobonenſi Univerſitate Profeſſoris Primarii, &c. Tomus tertius Rationis Medendi Continuatæ ſeu operum poſthumorum Volumen* I. *Collegit ediditque* Maximilianus Stoll, *S. C. R. Apoſtolicæ Majeſtatis Conſil. in eademque Univerſitate Profeſſor Praxeos P. O.* 8vo. Viennæ, 1779. p. 469.

DR. STOLL, the editor of this poſthumous collection, informs us in his preface, that he engaged in it at the requeſt of Baron Storck, to whom the late Dr. de Haen bequeathed his papers.

This

This first volume, which is ornamented with a good engraving of the author, is to be followed by two others that are to complete the work. The titles of the six sections, into which it is divided, are as follows: I. *Casuum variorum decas.* II. *Aphorismi de Diagnosi et Prognosi in acutis*, et *Hippocraticæ circa urinas doctrinæ compendium.* III. *Historia Morborum præcipue Variolarum anno* 1744 & 1745 *Hagæ-Batavorum observatarum.* IV. *De tussi puerorum convulsiva, finiente anno* 1746 & *initio* 1747 *Hagæ-Batavorum observata.* V. *Historia Medica annorum* 1747 & 1748 *Hagæ-Batavorum conscripta.* VI. *Dissertatio de colica pictonum, olim ab auctore edita, nunc vero multis in locis ab eodem auctore correcta, et casibus ægrorum ineditis locupleta.*

The subject of the first of the ten cases related in the first section was a married woman, aged 24, who from her infancy had been subject to cough. The chief symptoms of the disease were lassitude, head-ach, oppression at the breast, cough, quick pulse, fever, difficult deglutition, and at times pain in the abdomen. A minute journal is given of the case from the 19th of April to the 22d of the following month, when the patient died. An account is added of the appearances on dissection. The difficulty of swallowing was probably bably merely the effect of ſpaſm, as no cauſe of it could be diſcovered in the dead body. The lungs were found diſeaſed, and adhering to the pleura; and in the abdomen an introſuſception was obſerved in the inteſtinum ileum.

In the ſecond caſe we have an account of a *hernia femoralis* in a woman aged 59 years. The caſe terminated fatally, though the inteſtine was reduced fifteen hours before the death of the patient. The vomiting and ſingultus ceaſed immediately after the reduction of the hernia, but no ſtool followed it. On diſſection the hernia was found to have been occaſioned by a portion of the *inteſtinum ileum* which was free from obſtruction, and not in a diſeaſed ſtate. A pound of crude mercury, which had been given to the patient, was found in the ſtomach and duodenum, having been prevented from paſſing further by the preſſure of the omentum, which was much diſeaſed.

The third is the caſe of a ſhoemaker, who from his 22d to his 29th year had been troubled with ſymptoms of the ſtone, for which he was admitted into the hoſpital, where he remained under the author's care from Feb. 1756 to June 1757, when he was diſmiſſed cured. An account of his caſe is to be found in the

ſecond

ſecond and third volumes of the *Ratio Medendi*. From that time he remained perfectly free from any ſymptoms of calculus, but was ſubject to frequent attacks of pleuriſy, which at length proved fatal to him in October 1773. On diſſection no calculus was found in the bladder, but upon opening it, it appeared of a white colour, diſtended, rough, and as it were of a callous conſiſtence. Externally at the fundus of the bladder the peritonæum was reflected over an enormous maſs of fat.

The fourth caſe affords us the diſſection of a dropſical woman, of whoſe diſeaſe no account was to be found in De Haen's papers. The cutaneous veins of the neck and thorax were varicous, and on opening the abdomen a large cyſt was diſcovered adhering every where to the peritonæum, and containing about 24 quarts of a fœtid reddiſh water. A great part of the ſac was filled with a firm, fleſhy-like maſs, and in ſome places with a ſteatomatous and atheromatous ſubſtance divided into ſeveral ſmaller cyſts, in all of which there was the ſame kind of fluid as in the larger one. The body of the uterus was preſſed upwards by this tumour, and the *cervix uteri* ſtretched four inches in length. The tubes and ovaria were perfectly ſound.

The ileum was compreffed by the hard part of the tumour, and together with the jejunum was diftended to three inches in diameter. The omentum adhered to the anterior furface of the cyft, and its veins were fufficiently dilated to admit a goofe quill. The ftomach, liver, and fpleen were free from difeafe, but pufhed up higher than ufual.

The fifth was a cafe of what the author calls a *pleuro-peripneumonia*. The patient was a maid fervant, 24 years old. The difeafe began with painful refpiration, anxiety, colic pains, vomiting, and a ftrong, full, equal pulfe. The ftate of the pulfe continued the fame till within five hours of her death. The vomiting ceafed on the feventh day of the difeafe. Here the author takes occafion to inform us, that it was a general obfervation in the hofpital, that in acute difeafes the more the patients drink the lefs they vomit, (' *quo plus bibunt ægri, eo minus vomunt*'). On examining the dead body of this patient the cavities of the heart, aorta, and pulmonary artery were found filled with a polypous concretion. The lungs, pleura, and diaphragm were in a highly inflamed ftate.

The fixth is a cafe of jaundice fucceeded by dropfy, in a weaver twenty-five years old.

The

The difeafe terminated in death, and on diffection fixteen quarts of a yellowifh and fœtid water were found in the cavity of the abdomen. The liver was indurated, diminifhed in bulk fo as to weigh only a pound and nine ounces, and was in fome parts of a brownifh-yellow colour, and in others in a gangrenous ftate. The gall bladder was fmall, and its coats thickened. Its fundus was gangrenous, and it contained a dark red coloured fanies. Its neck was obftructed by a gall ftone, of an oval fhape, of about the fize of a fmall filberd nut. The ductus choledochus communis remained open, fo that the hepatic bile had a free courfe into the inteftine. The fpleen was harder and larger than ufual. The pancreas was of a cartilaginous confiftence, and compofed as it were of a great number of fmall tubercles. The lungs abounded with tubercles, fo as to exhibit nearly the fame appearance as the pancreas. The dura mater, periofteum, and ligaments had acquired a yellowifh colour, but the pia mater was lefs affected. The medullary fubftance of the brain was extremely white, and the cortex was likewife free from any yellow tinge.

In the fifth cafe we have an account of a rheumatic fever, of which the patient recovered.

 The

The feventh contains the diffection of a youth, thirteen years old, who was drowned in the Danube. On examining the brain of this fubject the veins of the pia mater, and the finufes of the dura mater at the bafis of the cranium, appeared greatly diftended with blood, but the arteries were in their natural ftate. The ventricles of the brain were empty. The confiftence of the cerebrum was extremely firm. The lungs were turgid with air, and fwam when thrown into water.

After this follows the diffection of a dropfical head. The fubject was a boy four years and a half old. Two years before his death he was obferved to be lefs lively and active than ufual, but in other refpects was thought to be in good health. By degrees his head was found to increafe in bulk in a greater proportion than the reft of his body, fo that during the laft year of his life he was obliged to reft it almoft conftantly on a pillow. His fenfes remained undifturbed to the laft, and he was never convulfed. At length he became more and more difpofed to fleep, and in this ftate expired quietly June 5th, 1775.

On meafuring the fize of the head, a tape carried from the forehead to the occiput was found to meafure two (Vienna) feet wanting an inch; and from the vertex to the chin two feet. Throughout the whole coronal future the bones were dilated a finger's breadth, but the fagittal and occipital futures were pretty well clofed.

Upon opening the cranium no fluid was obfervable between the bones and the dura mater, or between the latter and the pia mater. This laft membrane and the cerebrum appeared in a natural ftate, but the latter was not fo deeply furrowed as in healthy fubjects. The lateral ventricles were diftended to an enormous bulk. The quantity of water collected in them amounted to upwards of three pints. The *thalami nervorum opticorum* were fo compreffed by the weight of fluid as to be hardly perceptible. The third and fourth ventricles, though filled with water from their communication with thofe above, were not diftended.

The cerebellum externally was in a purulent ftate; internally it was compofed of a yellow, homogeneous cheefe-like fubftance, without the leaft veftige of the *arbor vitæ*.

The tenth and laft is the cafe of an afthmatic woman, who died of apoplexy in the fifth month of her pregnancy. On diffection the veins of the pia mater were found to be varicous, and the lungs confiderably infarcted.

With

With regard to the other ſections of the preſent volume we ſhall content ourſelves with having enumerated their contents. The ſecond volume of the work is to contain the moſt intereſting caſes that occurred to the author during his reſidence at the Hague, and in the laſt will be given his medical correſpondence with Van Swieten.

